# Automated diagnosis of chronic obstructive pulmonary disease using deep learning applied to electrocardiograms

**DOI:** 10.1016/j.ebiom.2025.106066

**Published:** 2026-01-03

**Authors:** Akhil Vaid, Jiya Sharma, Joy Jiang, Joshua Lampert, Ashwin Sawant, Edgar Argulian, Stamatios Lerakis, Pranai Tandon, Patricia Kovatch, Charles Powell, Charles B. Cairns, Girish N. Nadkarni, Monica Kraft

**Affiliations:** a Division of Data Driven Digital Medicine, Icahn School of Medicine at Mount Sinai, New York, NY, USA; b Charles Bronfman Institute for Personalized Medicine, Icahn School of Medicine at Mount Sinai, New York, NY, USA; c Seton Hall University, South Orange, NJ, USA; d Helmsley Electrophysiology Center, Mount Sinai Hospital, New York, NY, USA; e Samuel L Bronfman Department of Medicine, Icahn School of Medicine at Mount Sinai, New York, NY, USA; f Mount Sinai Fuster Heart Hospital, Icahn School of Medicine at Mount Sinai, New York, NY, USA; g The Catherine and Henry J. Gaisman Division of Pulmonary, Critical Care and Sleep Medicine, Icahn School of Medicine at Mount Sinai, New York, NY, USA; h The Department of Genetics and Genomic Sciences, Icahn School of Medicine at Mount Sinai, New York, NY, USA; i Drexel College of Medicine, Drexel University, Philadelphia, PA, USA

**Keywords:** Machine learning, Artificial intelligence, Ai-ecg, Electrocardiogram, Copd, Pulmonology, Spirometry

## Abstract

**Background:**

Chronic Obstructive Pulmonary Disease (COPD) is a leading cause of morbidity and mortality globally. Effective management hinges on early diagnosis, which is often impeded by non-specific symptoms and resource-intensive diagnostic methods. This study assesses the effectiveness of electrocardiograms (ECGs) analysed via deep learning as a tool for early COPD detection.

**Methods:**

We utilised a Convolutional Neural Network model to analyse ECGs for detecting COPD. The primary outcome was the accuracy of a new clinical COPD diagnosis as determined by ICD codes. Performance was evaluated using Area-Under-the-Curve (AUC) metrics derived by testing against ECGs from a set of holdout patients, ECGs from patients from another hospital, and ECGs of patients with COPD within the UK BioBank (UKBB).

**Findings:**

We analysed a total of 208,231 ECGs from 18,225 COPD cases, matched to 49,356 controls by age, sex, and race. The model exhibited robust performance across diverse populations with an AUC of 0⋅80 (0⋅80–0⋅80) in internal testing, 0⋅82 (0⋅81–0⋅82) in external validation and 0⋅75 (0⋅71–0⋅78) in the UKBB cohort. Subsequent analyses linked ECG-derived model predictions with spirometry data, and model explainability highlighted P-wave changes as indicative of COPD.

**Interpretation:**

AI-powered ECG analysis offers a promising path for early COPD detection, potentially facilitating earlier and more effective management. Implementing such tools in clinical settings could significantly enhance COPD screening and diagnostic accuracy, thereby improving patient outcomes and addressing the global health burden of the disease.

**Funding:**

This work was supported in part through the computational and data resources and staff expertise provided by Scientific Computing and Data at the 10.13039/100007277Icahn School of Medicine at Mount Sinai and supported by the Clinical and Translational Science Awards (CTSA) grant UL1TR004419 from the 10.13039/100006108National Center for Advancing Translational Sciences; and R01HL167050-02 from the 10.13039/100000050National Heart, Lung, and Blood Institute.

**Research in context**

**Evidence before this study**

We performed a case-insensitive search on PubMed and Google Scholar in November 2025 for the terms “ai-ecg copd”, and “deep learning copd”. Prior work in this domain is limited. The PubMed search yielded no results. A single Google Scholar result was identified: a 2023 IEEE conference paper which involved a total of 12 subjects (6 COPD, 6 controls) using continuous ECG recordings and reported ∼100% classification accuracy without discussion of data partitioning, external validation, or control for information leakage. More recently, AI-ECG models have been developed for pulmonary hypertension and general pulmonary disease classification, but none to our knowledge have used large, multi-cohort real-world ECG databases with longitudinal follow-up. No other peer-reviewed or population-scale studies applying AI to routine 12-lead ECGs for COPD detection were identified.

**Added value of this study**

This study is the first to show that deep learning applied to standard 10-s, 12-lead ECGs can accurately detect COPD across large, real-world patient cohorts. Using over 760,000 ECGs, we demonstrate robust performance across internal testing, an external hospital cohort, and the population-based UK Biobank. Unlike previous exploratory studies, our work incorporates extensive validation across demographically and temporally distinct populations, as well as subgroup analyses in patients with arrhythmias and those with documented smoking histories. We further show that model predictions increase as patients approach their clinical diagnosis and independently predict future COPD in survival analysis. Finally, explainability and spirometry correlation analyses link the model's predictions to physiologically plausible ECG features and objective measures of airflow limitation. Together, these findings establish ECG-based deep learning as a scalable, reproducible, and biologically grounded approach for COPD detection.

**Implications of all the available evidence**

Our findings demonstrate that AI-enhanced ECG interpretation can extend the diagnostic utility of one of the most widely available clinical tests for earlier COPD detection. ECGs are inexpensive, routinely collected, and accessible across diverse care settings including those with limited access to spirometry or chest imaging. As such, this approach offers a pragmatic path to earlier identification of patients with undiagnosed or subclinical disease. Earlier recognition may facilitate timely smoking cessation, targeted therapies, and pulmonary rehabilitation, potentially slowing disease progression and reducing healthcare burden. The strong association between model output and physiologic impairment suggests that ECG-based AI may complement, rather than replace, spirometry by flagging patients who warrant further pulmonary evaluation. As AI-ECG tools continue to expand across cardiovascular and systemic diseases, incorporating COPD detection could support integrated, opportunistic screening strategies in everyday clinical practice. Prospective evaluation will be important to understand real-world performance, workflow integration, and population impact.

## Introduction

Chronic Obstructive Pulmonary Disease (COPD) is a pervasive and progressive respiratory condition that affects the lungs, leading to severe limitation in airflow. According to the World Health Organisation, COPD is the third leading cause of death worldwide,^1^ highlighting its significant global health burden. The primary cause of COPD is prolonged exposure to harmful particles or gases, with tobacco smoke being the most common culprit.^2^ The disease is characterised by symptoms such as persistent cough, sputum production, and shortness of breath, which progressively worsen over time. COPD is diagnosed through lung function testing and can be managed, but not cured, with treatments focussing on symptom relief and quality of life improvement.^3^ As a primarily preventable condition, public health initiatives emphasise the importance of reducing exposure to known risk factors, particularly smoking cessation, to combat the rising prevalence and impact of COPD.

COPD also significantly impacts cardiac function, particularly the right side of the heart, a relationship well-documented clinically and in medical research. In patients with COPD, the increased airway resistance and chronic hypoxaemia lead to pulmonary hypertension, a condition characterised by elevated blood pressure in the pulmonary arteries.^4^ This increased pulmonary pressure places a significant strain on the right ventricle, the chamber of the heart responsible for pumping deoxygenated blood to the lungs.^5^ Over time, this strain can lead to right ventricular hypertrophy (an enlargement of the heart muscle) and ultimately right heart failure, a condition known as cor pulmonale.^6^ Symptoms of cor pulmonale include pedal oedema, fatigue, and worsening dyspnoea significantly diminish the quality of life in patients with COPD.^7^ The interplay between COPD and right heart failure highlights the need for careful cardiac monitoring in COPD management.^8^ This cardiac complication underscores the severity of COPD as a systemic disease with multi-organ implications.^9^

Currently, there exist no formal guidelines to screen for COPD in asymptomatic individuals,^10^ and mainstay investigations for diagnosis—spirometry or chest CT, are time, personnel, and economically intensive.^11^ Compounded by irregular standards of outpatient care, diagnosis may be delayed by several years even after initial onset of symptoms. This is a critical gap, since with early diagnosis, COPD can be managed effectively through lifestyle changes, medication, and pulmonary rehabilitation–leading to overall better patient outcomes.^12^^,^^13^ Such proactive approaches can significantly slow or stop disease progression,^14^ improve quality of life,^14^ and potentially reduce healthcare costs^15^ associated with advanced COPD. Moreover, earlier cessation of smoking in the context of a diagnosis of COPD has its own protective effects in prevention of cardiovascular disease^16^ and neoplasia.^17^

This set of circumstances raises the requirement of an easily available screening or diagnostic modality—a task for which the electrocardiogram (ECG) is very well suited. The ECG is a versatile, non-invasive, and low-cost investigation. It is ubiquitous and often forms the first point of contact between a patient and the investigative modalities found within a healthcare system, and ECG monitoring sensors can be integrated into wearable devices.

However, physicians largely depend on experience and clinical guidelines to interpret ECGs by identifying specific diagnostic patterns, mainly directed towards cardiac ischaemia and rhythm abnormalities. However, this reliance on guidelines also means that physicians might overlook or misinterpret ECG patterns that do not conform entirely to established norms. This limitation is particularly relevant in the context of diseases like COPD, wherein ECG manifestations^18^ might not be straightforward or may deviate from typical patterns seen in other cardiac conditions. Changes in pulmonary vasculature tend to first affect the right heart, and the signal may simply be overridden by the increased mass of the left heart. Some ECG patterns, especially those indicative of early or mild COPD, may be too subtle for human interpretation.

These conditions enable Artificial Intelligence (AI) models to play a transformative role for COPD management and triage. Deep learning, a subset of AI, has the capability to analyze complex datasets and identify patterns that might be imperceptible to the human eye without being given explicit directions. In the context of ECG interpretation, deep learning algorithms can be trained on vast datasets consisting of ECG signals paired to ground-truth diagnosis of COPD to recognise subtle deviations that may assist in earlier detection of COPD-related cardiac alterations, and quicker diagnosis.^19^^,^^20^

In this work, we develop and validate an approach testing whether Convolutional Neural Networks (CNNs) applied to ECGs can provide accurate diagnosis of COPD and how these findings are associated with changes in spirometry.

## Methods

### Data sources

We utilised data from the five hospitals of the Mount Sinai Health System: Mount Sinai Hospital, Mount Sinai Morningside, Mount Sinai West, Mount Sinai Beth Israel, and Mount Sinai Queens. These hospitals serve a demographically diverse population of patients within New York City. Data were taken for 2006–2023 from within the GE MUSE system that exports electrocardiograms as individual XML files containing raw waveforms. Finally, we collected ECGs from the United Kingdom BioBank^21^ (UKBB) along with the recorded diagnosis of COPD.

Additionally, we queried the electronic healthcare record to identify patients with documented smoking history. The results from this query included a unique patient identifier, number of pack years, as well as date and time of last contact with a provider.

For model development, ECGs from four Mount Sinai hospitals (Mount Sinai Hospital, Mount Sinai West, Mount Sinai Beth Israel, and Mount Sinai Queens) were pooled and divided into training and internal testing cohorts using a 90 to 10 patient level split, ensuring that no patient contributed ECGs to both sets. ECGs from Mount Sinai Morningside were held out entirely and used only as the first external validation cohort. ECGs from the UKBB formed the second external validation cohort. Analyses requiring smoking history were restricted to the subset of Mount Sinai patients in the internal testing cohort who had structured documentation of ten or more pack years.

Raw spirometry measurements including FEV_1_, FVC, and FEV_1_ divided by FVC, as well as bronchodilator reversibility when available, were obtained only for Mount Sinai patients and were used to correlate lung function with model predicted COPD probability. Cox proportional hazards analyses were performed only in the subset of Mount Sinai patients who had longitudinal ECG data, complete demographic information, and documented smoking history. No UKBB data were used for smoking specific analyses, spirometry analyses, or temporal analyses.

Sex was included as a matching variable when constructing control cohorts and as a covariate in subgroup and survival analyses. Sex information was obtained from the electronic health record and, for UK Biobank participants, from baseline assessment data. In both datasets, sex was self-reported by participants at the time of registration or clinical intake. To create the control cohorts, we identified individuals without a recorded COPD diagnosis from a larger pool of patients. We excluded anyone with a COPD diagnosis and then matched remaining patients to cases based on age (exact year match), sex, and mapped race according to U.S. Census race categories. Each case was required to have at least three eligible controls meeting these criteria. From this pool, we randomly selected three controls per case using a fixed random seed to ensure reproducibility. To maintain temporal alignment, we included only controls with available ECGs recorded prior to the corresponding case's diagnosis date.

### Ethics

This study was reviewed and approved by the Institutional Review Board of the Icahn School of Medicine at Mount Sinai (IRB reference number HRP-503R). This approval covered all data sources used in the study, including ECGs, electronic health record data, and spirometry from all five hospitals in the Mount Sinai Health System (Mount Sinai Hospital, Mount Sinai Morningside, Mount Sinai West, Mount Sinai Beth Israel, and Mount Sinai Queens). The requirement for written informed consent from participants was waived due to the retrospective nature of the study and the use of previously collected, de-identified data. The use of UK Biobank data was performed under the institution's existing UK Biobank approval and Material Transfer Agreement, and all analyses complied with UK Biobank's ethics and governance framework. The IRB granted a waiver of informed consent due to the retrospective use of de-identified data.

### Definition of outcomes

The primary outcome of this study was accuracy of a new diagnosis of COPD determined solely by ECG. We considered ECGs within the six months prior to the first recorded diagnosis of COPD by ICD code.^22^ Patients were excluded if they had prior suspicion of COPD as demonstrated by use of inhaled bronchodilators or documentation of previous pulmonary function testing. Any ECG so collected was considered a case and associated with a positive label. For construction of the control cohort, we matched each patient to approximately three patients without COPD on the basis of age, sex, and U.S census-based race.

Since ICD codes are recorded for billing purposes and may not reflect disease progression, an additional validation cohort was created for the subset of patients in testing data with a documented smoking history of 10 or more pack years. As before, cases within this cohort were matched to approximately three controls along the same criteria.

ECGs taken from the UKBB were matched to three controls based only on age, and sex given the racial homogeneity of the UKBB cohort.

### Data preprocessing

The quality of the ECG recording is susceptible to degradation due to patient/electrode movement (baseline drift), interference due to action potentials in skeletal muscle (myogenic interference), or leakage of the Alternating Current required to run the recording apparatus into the recording (power-line interference). To account for these sources of noise, we performed filtering on raw waveform data through sequential application of a Butterworth bandpass filter to remove extraneous frequencies, followed by a median filter to correct for baseline drift.

We converted the raw ECG waveforms into 2D images for analysis using a convolutional neural network. While this transformation represents an artificial step since ECGs are natively stored as 1D signals, it also enables compatibility with many clinical systems that archive ECGs in image or PDF format, enhancing generalisability across institutions. We elected this approach to leverage the extensive body of research and well-optimised architectures in computer vision, which can provide robust performance even with limited data via transfer learning. Moreover, this format allows the use of established explainability techniques such as Grad-CAM, offering insights into which regions of the ECG image contribute to the model's predictions. Prior work by our group and others has demonstrated the effectiveness of this strategy for ECG-based classification tasks.^20^

### Model selection and training

We elected to utilise the ConvNeXt Large^23^ models because of their established accuracy and efficiency when dealing with data at scale. As a modern convolutional neural network designed to rival transformer-based architectures, ConvNeXt achieves strong accuracy while maintaining architectural efficiency. Its success in natural image classification tasks made it a strong candidate for modelling ECG waveforms represented as images. The model was trained to classify natural images into ∼22,000 different categories within a large 14 million image dataset. By utilising such models as starting points for other computer vision tasks (such as ECG based diagnosis), it becomes possible to achieve better performance with relatively less data. This technique is known as *transfer learning*^24^ and it has been shown to improve modelling across a wide variety of ECG based modelling problems.

We trained the CNN for a total of 25 iterations (epochs) through the training data with the Adam optimiser, an adaptive learning rate going from a minimum of 1e-3 to a maximum of 3e-4 as per the OneCycle policy, a batch size of 128, and Cross-Entropy Loss with mixed-precision (16-bit) enabled. To avoid overfitting, model performance was evaluated after each epoch on a smaller set of ECGs held out from the training cohort. A snapshot of the model was saved after each epoch, and the model with the best performance on this holdout set was used for further evaluation on the testing/external validation cohorts.

### Statistics

We assessed model performance at classification of the diseased vs non-diseased state was performed using Area Under the Curve metrics, i.e. Area Under the Receiver Operating Characteristic curve (AUROC) which relays the performance of a model at being able to differentiate positives from negatives. Values for AUROC range between 0⋅5 and 1, with higher values indicating better performance. We also utilised the Area Under the Precision Recall curve (AUPRC) with values ranging between the prevalence of the positive class and 1, and higher values indicating better performance.

Interpretation of AUROC and AUPRC varies slightly in that the baseline value of AUROC is, by convention, 0⋅5. Predictions from a model at this level of performance are no better than a random coin toss, and values substantially higher than 0⋅5 indicate better performance. Conversely, the baseline value of AURPC varies by the prevalence of the positive class in the dataset. For example, a dataset with 20% positives would have a baseline AURPC of 0⋅2. Thus, when considering AUPRC as a metric, values substantially greater than the recorded prevalence indicate better performance.

We also examined both the average probability output by the model for cases that were found to be eventually have COPD; as well as ROC curves derived against an equivalently sized randomly selected cohort of patients at timeframes outside the 6-month interval from time of clinical diagnosis. Such an analysis helps understand the utility of screening tools in real-world settings with no fixed timeframe of clinical diagnosis and underscores the relationship between pulmonary pathology and cardiac response. As a follow-up analysis, we applied a Cox Proportional Hazards approach to a smaller subset of patients in the testing cohort for whom historic ECG data were available alongside complete data of their smoking status and demographics. This model was fit to patient sex, age, pack years at time of ECG collection, and probability of COPD as output by the model. This approach yielded Hazard Ratios, which signify which of the predictors best estimate the risk of an adverse diagnosis.

Additionally, we implemented metrics that require a classification threshold, namely sensitivity, and specificity. All models output probability values of the positive class. Threshold dependent metrics rely upon a pre-determined cutoff value for discriminating between data points the model considers positives, and those the model considers negative. All values reported in this work assume a sensitivity of 85% for calculation of the classification threshold, and subsequent downstream utilisation of the model as a screening tool. Confidence intervals for all metrics were generated through 500-fold bootstrapping.

Deep learning models used as classifiers output the probability of any one sample or datapoint belonging to a class or category. We correlated these probabilities to respiratory parameters derived from spirometry using the Pearson correlation coefficient.^25^ Correlation coefficients range between −1 and +1, with values less than 0 implying negative correlation, and values greater than 0 implying positive correlation. In this context, negative correlation indicates the magnitude of the dependent variable will reduce with increases in the independent variable. Additionally, absolute values of the coefficient between 0 and 0⋅5 imply weak correlation, while absolute values between 0⋅5 and 1 imply strong correlation.

### Software and hardware

We utilised the pandas, polars, numpy, connectorx, PyTorch, torchvision, scikit-learn, scipy, statsmodels, matplotlib, and seaborn libraries within the Python programming language (3⋅8⋅x). We generated saliency maps using the FullGrad implementation within Grad-CAM, which aggregates gradient and bias information across all layers of the model to produce comprehensive input attributions. FullGrad was applied to the trained ConvNeXt Large model without requiring manual selection of a specific convolutional layer. The resulting heatmaps were overlaid on the original ECG images to visualise the regions that contributed most to the model's prediction of COPD. All code was run on the Minerva HPC cluster at Mount Sinai.

### Role of funders

Funding entities had no role in the study design, data collection, data analysis, interpretation of data, or writing of the report.

## Results

**Fig. 1:**
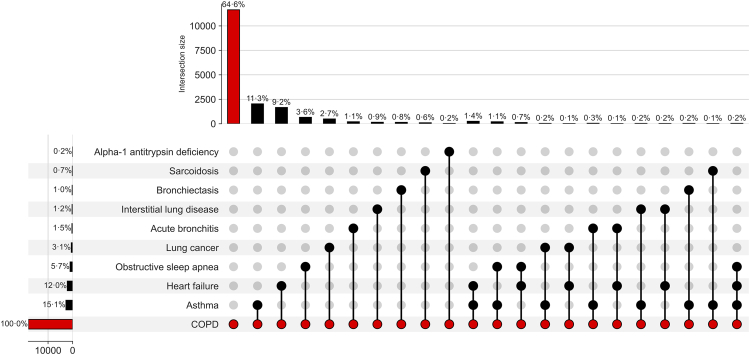
**Additional diagnoses for patients in training cohort (n = 18,225 patients)**. UpSet plot depicting the frequency and co-occurrence structure of other ICD-code based respiratory and cardiopulmonary diagnoses present within 30 days of the COPD index date in the training set. The horizontal bars on the left show the number and percentage of COPD patients with each individual diagnosis. Vertical bars at the top indicate the intersection sizes, i.e. the number of patients with specific combinations of these additional diagnoses occurring together with COPD. Each column of connected filled circles denotes one unique combination of coexisting conditions; only intersections representing ≥0⋅1% of the COPD cohort (≥18 patients) are shown to avoid overplotting. Red circles represent COPD (present in all patients), while black circles indicate additional diagnoses contributing to that specific intersection.

We collected 208,231 ECGs from 18,225 patients diagnosed with COPD, matched to 49,356 controls with 552,771 ECGs, for a total of 761,002 ECGs. With these data, we trained a CNN based on the ConvNeXt Large architecture and tested its performance on three cohorts i.e. an internal testing cohort, an external validation cohort with patients from Mount Sinai Morningside, and an additional external validation cohort with 258 COPD cases, and 1290 age and sex matched controls from the UKBB. Additionally, we queried the electronic healthcare record for patients with recorded smoking history of 10 or more pack years who were also in the internal testing cohort. Within this cohort, we located 4293 patients with 57,053 ECGs. Importantly, this smaller cohort represents the patients located through database queries on EHR reporting tables containing smoking history. Demographic characteristics of all cohorts are as enumerated in Table 1. Of the 18,225 patients identified with COPD, 64⋅6% had COPD alone, with the remainder also having COPD and other diagnoses, including asthma (15⋅1%), heart failure (12%), obstructive sleep apnoea (5⋅7%), lung cancer (3⋅1%) and acute bronchitis (1⋅5%) as the most common (Fig. 1).

**Table 1:** Demographic characteristics of study populations.

	Internal testing		External validation (Morningside)	
	Cases	Controls	Cases	Controls
Patients	18,225	49,356	3430	10,252
ECGs	208,231	552,771	24,286	84,206
Male	8838 (48%)	23,816 (48%)	1547 (45%)	4909 (48%)
Female	9369 (52%)	25,393 (52%)	1878 (55%)	5313 (52%)
Age (std)	67⋅49 (13⋅10)	67⋅68 (12⋅97)	66⋅40 (12⋅89)	66⋅64 (13⋅30)
Race				
Unknown/Other	8759 (48%)	17,216 (35%)	1715 (50%)	3340 (33%)
White	5596 (31%)	20,271 (41%)	738 (22%)	2365 (23%)
African American	2035 (11%)	9613 (19%)	688 (20%)	4088 (40%)
Hispanic	1658 (9%)	1195 (2%)	273 (8%)	355 (3%)
Asian	177 (1%)	1061 (2%)	16 (0⋅5%)	104 (1%)
Spirometry				
Patients	4058	–	629	–
Studies	9861	–	957	–
FEV_1_ [Mean (STD)]	1⋅47L (0⋅62)	–	1⋅52L (0⋅63)	–
Predicted FEV_1_ [Mean (STD) <%>]	2⋅46L (0⋅60) <59⋅8%>	–	2⋅45L (0⋅56) <62⋅0%>	–
FVC [Mean (STD)]	2⋅39L (0⋅88)	–	2⋅41L (0⋅86)	–
Predicted FVC [Mean (STD) <%>]	3⋅19L (0⋅79) <74⋅9%>		3⋅18L (0⋅75) <75⋅8%>	
FEV_1_/FVC [Mean (STD)]	0⋅62 (0⋅15)	–	0⋅64 (0⋅21)	–

### Performance for COPD diagnosis

Performance was maintained across all three cohorts, with AUROC values of 0⋅80 (95% CI: 0⋅80–0⋅80) in internal testing, 0⋅82 (95% CI: 0⋅81–0⋅82) in external validation in the Morningside cohort, and 0⋅75 (95% CI: 0⋅71–0⋅78) in the UKBB cohort.

**Fig. 2:**
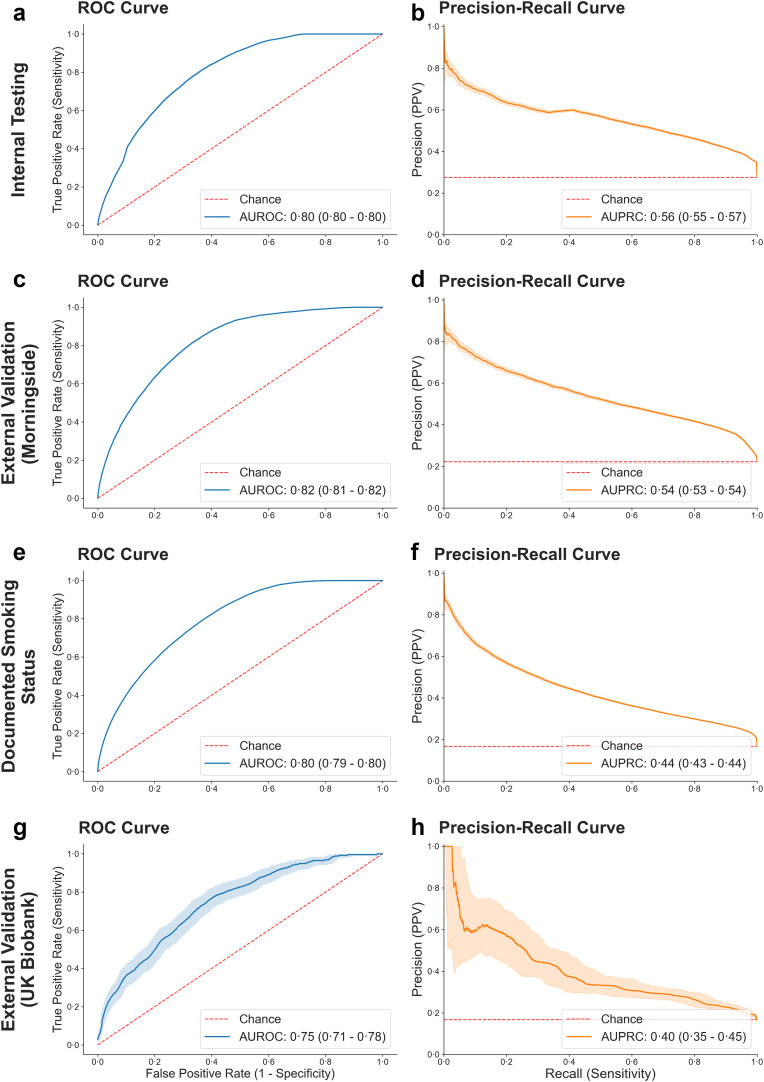
**Performance curves**. Panels show classification performance of the CNN model using ECGs from: (a–b) the internal testing cohort (182,231 COPD-positive ECGs; 552,771 control ECGs), (c–d) the Mount Sinai Morningside external validation cohort (24,286 COPD-positive ECGs; 84,206 controls), (e–f) the subset of patients with a documented smoking history of ≥10 pack-years (57,053 ECGs from 4293 patients), and (g–h) the UK Biobank external validation cohort (258 COPD cases; 1290 matched controls). Panels a, c, e, g show ROC curves with corresponding AUROC values and 95% confidence intervals generated via 500-fold bootstrapping. Panels b, d, f, h show precision–recall curves (PRCs), plotted with the same bootstrapping procedure. Dashed red lines represent performance expected by random chance for each metric (0⋅5 for AUROC; prevalence-matched baseline for AUPRC).

For the smaller subset of patients with a known smoking history of 10 or more pack years, model performance was found to be equivalent to the overall cohort based on ICD codes with AUROC values of 0⋅80 (95% CI: 0⋅80–0⋅80) (Fig. 2, Table 2).

**Table 2:** Model performance.

	AUROC	AUPRC	Specificity	PPV	NPV
Internal testing	0⋅80 (0⋅80–0⋅80)	0⋅56 (0⋅55–0⋅57)	0⋅59 (0⋅58–0⋅60)	0⋅44 (0⋅43–0⋅44)	0⋅91 (0⋅91–0⋅91)
External Validation (Morningside)	0⋅82 (0⋅81–0⋅82)	0⋅54 (0⋅53–0⋅54)	0⋅63 (0⋅63–0⋅64)	0⋅40 (0⋅39–0⋅40)	0⋅94 (0⋅94–0⋅94)
Smokers ≥ 10 pack years	0⋅80 (0⋅80–0⋅80)	0⋅44 (0⋅43–0⋅44)			
External Validation (UKBB)	0⋅75 (0⋅71–0⋅78)	0⋅40 (0⋅35–0⋅45)	0⋅47 (0⋅39–0⋅56)	0⋅24 (0⋅22–0⋅28)	0⋅94 (0⋅93–0⋅95)

AUROC and AUPRC are threshold independent metrics. Sensitivity, Specificity, PPV, and NPV are threshold dependent metrics. All threshold dependent metrics assume a sensitivity of 0⋅85.

Similar trends were seen for AUPRC, with values of 0⋅56 (95% CI: 0⋅55–0⋅57) against a prevalence of 0⋅28 in internal testing, 0⋅54 (95% CI: 0⋅53–0⋅54) against a prevalence of 0⋅22 in Morningside patients, 0⋅44 (0⋅43–0⋅44) against a prevalence of 0⋅18 within the cohort of smokers of 10 or more pack years. Finally, AUPRC was 0⋅40 (95% CI: 0⋅35–0⋅45) against a prevalence of 0⋅17 in the UKBB cohort (Fig. 2, Table 2). Model performance was consistent across sexes, with comparable AUROC, AUPRC, specificity, PPV, and NPV for male and female patients (Supplementary Table S1).

To further address concerns that model performance may be confounded by the presence of underlying arrhythmias, we stratified evaluation by major arrhythmia categories. Across six clinically distinct arrhythmia groups (including atrial arrhythmias, ventricular arrhythmias, conduction abnormalities, junctional rhythms, paced rhythms, and accessory pathways), the model maintained robust performance. AUROC values ranged from 0·77 to 0·81, with overlapping 95% confidence intervals across subgroups (Supplementary Table S2, Supplementary Figure S2), indicating that model performance was not disproportionately driven by any single arrhythmia type. This suggests that the model's discriminative ability generalises beyond rhythm-specific features and supports its capacity to detect COPD-relevant patterns even in the presence of varying underlying cardiac rhythms.

**Fig. 3:**
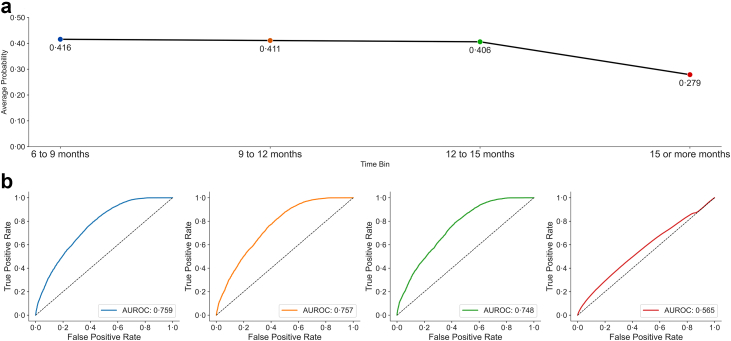
**Evolution of model performance**. Panel a shows the mean uncalibrated model-predicted COPD probability for ECGs obtained from COPD-positive patients at increasing temporal distances from the first recorded diagnosis, grouped into 6–9 month, 9–12 month, 12–15 month, and ≥15 month bins. Model outputs decreased as ECGs were sampled further away from the date of clinical diagnosis. Panel b displays the AUROC for each time bin, calculated by comparing COPD-positive ECGs to an equivalently sized randomly selected control population for each interval. Discriminative performance declined from AUROC 0⋅76 at 6–9 months prior to diagnosis to AUROC 0⋅57 for ECGs obtained ≥15 months before diagnosis.

When considering ECGs collected outside the 6-month time interval for patients with a diagnosis of COPD, model performance increased in inverse proportion to the time between collection of the ECG, and time of diagnosis. When the model was applied to these cases, model confidence at positive diagnosis was found to increase closer to the time of clinical diagnosis, with a maximal value of 0⋅42 if considering ECGs between 6 and 9 months from the date of diagnosis. This value dropped to 0⋅28 when considering ECGs which were collected at or more than 15 months prior to the date of diagnosis. Similarly, AUROC values measured against an equivalently sized, randomly collected set of control samples were at 0⋅76 at 6–9 months out, dropping mildly over the course of the next 9 months, and then to nearly random (0⋅57) at or more than 15 months from time of clinical diagnosis (Fig. 3).

**Fig. 4:**
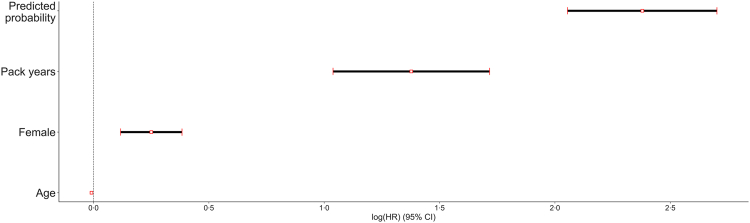
**Cox Proportional Hazards Model**. The analysis included 518 patients with 1321 historical ECGs and complete demographic and smoking data. The hazard ratio for model-predicted COPD probability was 10⋅79 (95% CI: 7⋅81–14⋅91), while pack-years at the time of ECG showed an HR of 3⋅96 (95% CI: 2⋅83–5⋅56). Female sex was associated with an HR of 1⋅28 (95% CI: 1⋅13–1⋅47), and age demonstrated a slightly protective effect with an HR of 0⋅99 (95% CI: 0⋅99–1⋅00). Sex was coded as Male = 0 and Female = 1. Hazard ratios reflect the exponentiated Cox model coefficients. Hazard ratios are calculated by taking by taking the exponent of the (log) values on the x-axis.

In an additional analysis using the Cox Proportional Hazards model on a subset of 518 patients with 1321 historical ECGs from the start of smoking to the time of COPD diagnosis, we found that the model's uncalibrated probability output was strongly associated with an eventual COPD diagnosis, with a hazard ratio of 10⋅79 (95% CI: 7⋅81–14⋅91, P < 0⋅005 [Cox proportional hazards model]). For comparison, the number of pack years at the time of ECG collection had a hazard ratio of 3⋅96 (95% CI: 2⋅83–5⋅56, P < 0⋅005 [Cox proportional hazards model]), and being female was associated with a hazard ratio of 1⋅28 (95% CI: 1⋅13–1⋅47, P < 0⋅005 [Cox proportional hazards model]) (Fig. 4, Table 3).

**Table 3:** Cox Proportional Hazards model summary.

Covariate	Coefficient (coef)	Hazard ratio (exp (coef))	Coef⋅ lower 95% CI	Coef⋅ upper 95% CI	HR lower 95% CI	HR upper 95% CI	z-value	P-value [Cox PH]
Pack years	1⋅38	3⋅96	1⋅04	1⋅72	2⋅83	5⋅56	7⋅99	<0⋅005
Age	−0⋅01	0⋅99	−0⋅01	0	0⋅99	1	−3⋅22	<0⋅005
Female	0⋅25	1⋅28	0⋅12	0⋅38	1⋅13	1⋅47	3⋅72	<0⋅005
Predicted probability	2⋅38	10⋅79	2⋅06	2⋅7	7⋅81	14⋅91	14⋅42	<0⋅005

### Explainability of model predictions

**Fig. 5:**
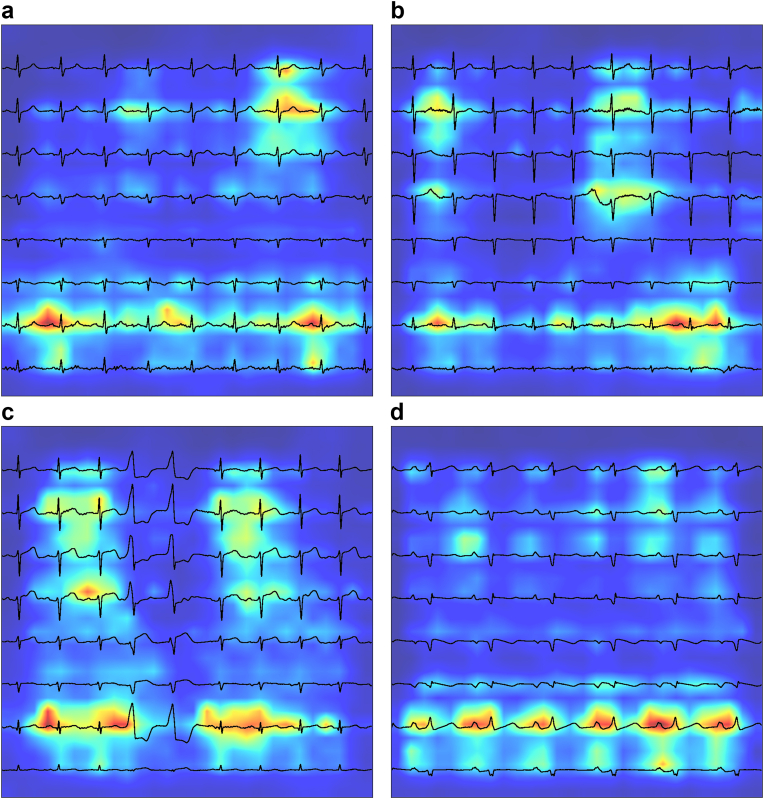
**Explainability plots**. Saliency maps generated using FullGrad, applied to the trained ConvNeXt Large model, are overlaid on four representative 12-lead ECGs from distinct patients (a–d). Regions highlighted in warmer colours (yellow to red) indicate areas contributing most strongly to the model's prediction of COPD. Across examples, attribution predominantly focuses on P-wave morphology and contiguous atrial segments, consistent with cardiac manifestations of COPD-related pulmonary vascular remodelling.

We created heatmaps which highlighted the regions of the ECG most responsible for pushing model predictions in the direction of a positive diagnosis of COPD. These heatmaps were overlaid on top of the ECG proper and plotted to images as shown in Fig. 5.

We found that the model tended to focus on P waves for patients with COPD. In the shown example, bifid P waves were considered most relevant by the model towards deciding on a positive diagnosis of COPD.

### Correlation between model predictions and spirometry parameters

We collected spirometry for 8280 patients paired to 54,647 ECGS. We also collected spirometry for 2181 patients with documented irreversible airflow obstruction paired to 9909 ECGs. In both cases, these pairs of investigation results were restricted to patients with FEV_1_/FVC ratios of ≤ 0⋅7, and ECGs which were collected within 6 months of the date of the Spirometry (Supplementary Figure S1). Predictions were made on collected ECGs, and the probability of the patient having COPD according to the model was correlated to values of predicted FEV_1_%, predicted FVC%, and predicted FEV_1_/FVC as defined by the percentage ratios between measured and predicted values of these parameters.

**Fig. 6:**
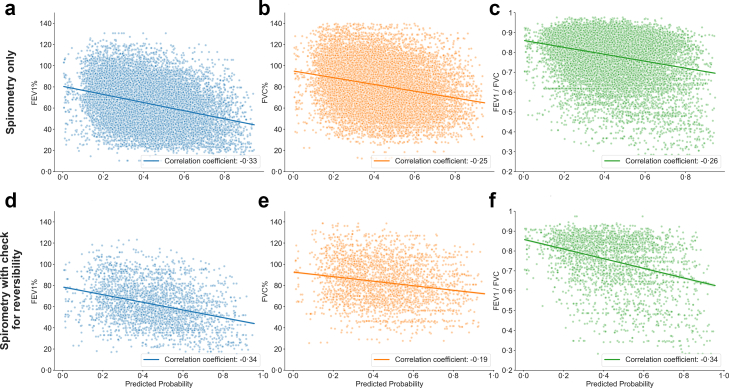
**Correlation between predicted probability of COPD and % predicted FEV_1_, % predicted FVC, and FEV_1_/FVC**. Scatterplots show the relationship between model-predicted COPD probability and spirometric indices expressed as percent predicted using GLI-Global reference equations. Panels a–c include 54,647 ECG-spirometry pairs with FEV1/FVC ≤ 0⋅70 and no bronchodilator reversibility testing; panels d–f include 9909 ECG-spirometry pairs and confirmed irreversible obstruction. Correlation coefficients were −0⋅33 and −0⋅34 for FEV1%, −0⋅25 and −0⋅19 for FVC%, and −0⋅26 and −0⋅34 for FEV1/FVC in the spirometry-only and irreversibility cohorts, respectively, indicating that higher predicted probability is associated with more impaired lung function. Each point represents a down sampled ECG-spirometry pair to reduce overplotting. Consistent negative correlations indicate that higher AI-predicted probability of COPD is associated with more impaired lung function.

We found that there was an overall weakly negative correlation between model predictions and the values of the parameters measured. In the group of patients with the reduced FEV_1_/FVC ratio and no further testing about bronchodilator mediated reversibility, correlated coefficients were −0⋅33, −0⋅25, and −0⋅26 for FEV_1_, FVC, and FEV_1_/FVC, respectively. These values because more strongly negative for patients with reduced FEV_1_/FVC ratio and documented irreversibility of airflow obstruction with bronchodilators—reaching values of −0⋅34, −0⋅19, and −0⋅34 for FEV_1_, FVC, and FEV_1_/FVC, respectively (Fig. 6).

## Discussion

In this study, we leveraged the fact that COPD can affect the pulmonary vasculature to develop a novel ECG based deep-learning model that can identify patients who have COPD or are at risk of developing COPD. We externally validated this model on two separate populations and demonstrated the correlation between model predictions and measurements derived through spirometry. We further analysed subgroups of patients with known smoking histories, finding that model performance was maintained, and that model outputs were associated with the highest hazard ratio of an eventual diagnosis of COPD. Finally, we derived explainability to demonstrate that the model is indeed looking at specific regions of the ECG to make its decisions.

Isolated internal testing cannot guarantee model quality since the biases accruing from similarities of demographics, socioeconomic factors, and hospital processes may cause the algorithm not to generalise to other sites. We found that performance was maintained across all three tested patient subpopulations, including one cohort from a geographically separate region. We also tested the model in a subgroup of patients with well-documented smoking histories. Here, the model's predictions were strongly associated with an eventual COPD diagnosis, even more so than smoking history itself, suggesting it is capturing real physiological signals rather than just patterns linked to administrative codes. This is a reassuring finding and falls in line with the FDA's guiding principles for development of machine learning algorithms.^26^ In a similar vein, ICD codes, while widely used, are known to have systemic issues both in terms of over and underdiagnosis.^27^ Unfortunately, such biases can compromise the validity of studies relying solely on these codes for disease identification. Additionally, the maintenance of model performance in a subpopulation of patients with a well-documented smoking history is particularly significant. This suggests that the model is accurately identifying the relevant physiological signal associated with COPD, beyond the limitations of ICD code reliability. This validation within a more precisely characterised group indicates that out model is likely detecting the right underlying factors. Lastly, the additional diagnoses present with COPD in 36⋅4% of the cohort (Fig. 1) are not surprising and often do coexist with COPD. This finding also suggests that other obstructive lung diseases may be uncovered with this approach.

Our analysis of spirometry-paired ECGs revealed negative correlations between model-predicted probability of COPD and FEV1%, FVC%, and FEV1/FVC (Fig. 6), suggesting that the model is more likely to detect COPD in individuals with greater physiologic impairment. These findings imply that the model may be less sensitive to early or mild disease, where pulmonary vascular and right heart changes may not yet be present or discernible on ECG.

Threshold selection has an important part to play in the real-world usage of such algorithms.^28^ At an appropriately low threshold (with resulting high sensitivity), deep learning models may be utilised as “screening” tools. In the absence of established screening guidelines for COPD, such tools can assist in earlier identification of pathology and subsequently better outcomes. Further, the decision to run or prioritise confirmatory testing on top of the screening may be delegated to on-the-ground clinicians through the use of calibrated probabilities or decision curves^29^ in addition to, or instead of binary low/high risk prediction.

Correlation of model predictions to spirometry results further illuminate the pathophysiology of COPD. The ECG detects changes in the myocardium secondary to changes in the pulmonary vasculature. Therefore, detection of COPD using deep learning considers the cumulative myocardial damage and remodelling up to the point of measurement. Thus, detection of COPD by ECG may be better for individuals with lower cardiac resilience, or poorer control despite the duration or severity of disease. Consistent with this, our model was more robust closer to the diagnosis and patient demonstrated significant airflow limitation with FEV_1_/FVC <0⋅7 and % predicted FEV_1_ ranging between 59 and 62% across the two cohorts. While the model demonstrates the ability to identify patients months before a formal COPD diagnosis (Fig. 3), this likely reflects the presence of undiagnosed or subclinical disease already producing physiologic changes. The observed decline in model performance beyond 15 months supports this interpretation, suggesting that the model is not predicting new-onset COPD in healthy individuals, but rather flagging patients with early pathophysiologic alterations not yet captured by formal diagnosis. Future longitudinal studies starting from a baseline of normal spirometry would be necessary to evaluate the model's potential for true prediction of disease onset.

Importantly, the utility of this model does not hinge on the precise disease stage at the time of formal diagnosis. By design, we analysed ECGs collected prior to the first recorded diagnosis of COPD, meaning that our model demonstrated predictive signal before the clinical recognition of the disease, regardless of its severity. Given that ECGs are often obtained for other indications in hospital or emergency settings, this creates an opportunity for opportunistic case-finding. If model predictions had been available at the time of ECG collection, the diagnosis of COPD could potentially have been made earlier, enabling earlier treatment or referral to pulmonary care. This highlights the role of AI-based ECG analysis not as a replacement for spirometry, but as a screening aid to accelerate diagnosis, particularly in the many cases where delays in clinical recognition occur.

From a practical standpoint, AI-ECG models are well-suited for real-world deployment in hospital settings. The electrocardiogram is a universally available, low-cost investigation that is already embedded in routine clinical workflows across inpatient, emergency, and outpatient care. This fact facilitates integration into hospital infrastructure without the need for specialised hardware or new ECG acquisition protocols. As AI-based ECG interpretation becomes more commonplace in clinical practice, our approach offers a feasible pathway to extend these capabilities to respiratory disease, particularly in the context of opportunistic screening for COPD. Before broader use, we plan to pilot the model in a small number of hospitals to see how it performs in everyday workflows, measure its impact on time to diagnosis, and address operational considerations such as processing speed, scalability, and ensuring equitable performance across patient groups.

Major strengths of this study include the large and diverse dataset, comprising over 760,000 ECGs from more than 67,000 patients across five hospitals in the Mount Sinai Health System, supplemented by an independent population-based cohort from the UK Biobank. The inclusion of multiple, demographically distinct populations increases the generalisability of the findings. Additionally, the use of real-world ECGs acquired in routine care without specialised protocols supports clinical integration, and the multi-cohort validation strategy demonstrated stable performance across internal, hospital-based, and population-based cohorts. Linking ECG predictions to spirometry-derived lung function metrics provided physiologic validation, while explainability analyses highlighted ECG regions plausibly associated with COPD-related cardiac changes. Finally, the model also retained discriminative ability up to 15 months before diagnosis, suggesting utility for identifying subclinical disease or early pathophysiologic changes before formal recognition.

This study should be interpreted in light of some limitations. The performance of our ECG-based model could potentially be enhanced by incorporating patients' smoking status, a known risk factor for COPD. However, the modelling itself did not include smoking data due to unavailability of reliable smoking history for all patients included within the study. Indeed, we were able to locate smoking history for only a small subset of patients present within the overall cohort—potentially due to limitations in how these data were catalogued within the EHR. However, we were encouraged to find that model performance was maintained for the subset of patients for whom a reliable smoking history was available. Additionally, we only included ECGs within 6 months of diagnosis for training the model. While the model demonstrated strong performance within the 6-month timeframe, its accuracy diminishes beyond this period, particularly after 15 months. Detection up to 15 months earlier than the standard approach could be beneficial, as it could allow for timely interventions. It is important to acknowledge, though, that increased sensitivity in screening for earlier detection could lead to a higher rate of false positives, which might result in unnecessary follow-up tests or treatments. However, the Cox proportional hazards model applied to historical ECG data for smokers eventually diagnosed with COPD showed that model predictions are even more strongly associated with an eventual diagnosis of COPD than the smoking history itself. Another limitation is the potential confounding effect of comorbid conditions such as heart failure or atrial fibrillation, which may also influence ECG morphology; while these were not explicitly adjusted for, model performance remained consistent in both general and smoking-specific cohorts, suggesting it captures COPD-relevant signals. Further, we note that explainability results derived from saliency maps, including the emphasis on P-wave regions should be interpreted with caution. These maps reflect correlational attribution and are not evidence of causal relationships. Moreover, given the known clinical overlap between COPD and arrhythmias such as atrial fibrillation, the model's focus on atrial electrical activity may partly reflect comorbid patterns. Stratified analyses by arrhythmia subtype, along with more mechanistic and statistically driven investigations into COPD-related ECG changes, represent important directions for future work. Regardless, it would be interesting to determine if the combination of ECG analysis and smoking history would enhance the ability to predict early COPD, and we consider this an avenue for future work.

In conclusion, we found that the utilisation of AI to utilise ECGs for the screening for or diagnosis of COPD in heterogenous populations represents an important step forward in early identification and arresting disease progression. Further exploration of this non-invasive, widely available technology as a low-cost screening tool appears warranted.

This study developed a deep learning model that analyzes electrocardiograms (ECGs) to identify people who have or are at risk for chronic obstructive pulmonary disease (COPD). The model was trained on over 760,000 ECGs from 67,000 patients across five hospitals, plus an independent UK Biobank cohort.

The model's predictions were validated externally and shown to correlate with spirometry (lung function) results, meaning it captures real physiological changes associated with COPD. It also performed well in subgroups with known smoking histories, indicating it detects underlying disease signals rather than relying on administrative codes or smoking data alone. Explainability analyses confirmed the model focused on specific ECG regions consistent with COPD-related cardiac changes. Performance was strongest near the time of diagnosis, suggesting it identifies undiagnosed or early disease, not future new-onset cases.

As ECGs are widely available and low-cost, this AI-based approach could serve as a practical screening tool to flag patients for earlier testing and diagnosis, complementing but not replacing spirometry.

## Contributors

AV acquired data, performed statistical analysis, and draughted the manuscript. AV, GNN, and MK conceived and designed the study. AV, JS, CBC, and MK analysed and interpreted the data. MK and GNN supervised the study and contributed to data acquisition. JS, JJ, JL, AS, EA, SL, PT, CP, CBC, and PK critically revised the manuscript for important intellectual content. PK provided administrative, technical, and material support for high-performance computing resources. AV and GNN had access to and verified the data. All authors reviewed and approved the final version of the manuscript and made the decision to submit for publication.

## Data sharing statement

ECG data from the MSHS will not be released because of patient privacy implications. Data from the UK BioBank is publicly available upon appropriate request to the relevant administering authorities. Code for training and testing the models developed within the study is available at https://github.com/akhilvaid/ECGCOPD under a GPLv3 licence.

## Declaration of interests

Dr. Nadkarni reports consultancy agreements with AstraZeneca, BioVie, GLG Consulting, Pensieve Health, Reata, Renalytix, Siemens Healthineers, and Variant Bio; research funding from Goldfinch Bio and Renalytix; honoraria from AstraZeneca, BioVie, Lexicon, Daiichi Sankyo, Meanrini Health and Reata; patents or royalties with Renalytix; owns equity and stock options in Pensieve Health and Renalytix as a scientific cofounder; owns equity in Verici Dx; has received financial compensation as a scientific board member and advisor to Renalytix; serves on the advisory board of Neurona Health; and serves in an advisory or leadership role for Pensieve Health and Renalytix. Dr. Lampert reports a consultancy agreement with Viz.ai. Dr. Vaid reports a consultancy agreement with Verily Inc and HeartSciences Inc. Dr. Cairns reports grants or contracts from the National Institutes of Health and the Bill & Melinda Gates Foundation (funds paid to Drexel University); consulting fees from bioMérieux (consultant for biomarker development until 2023, funds paid personally); participation on Data Safety Monitoring Boards and advisory boards for the National Institutes of Health (DSMB, NHLBI COVID-19 Convalescent Plasma of Outpatients) and Respond Health (Scientific Advisory Board, unpaid); and leadership or fiduciary roles with several non-profit organisations, including the American Association of Medical Colleges (Administrative Board, Council of Deans), St. Christopher's Hospital for Children (Board Member), Society for Academic Emergency Medicine Foundation (Board Member), Eureka Institute for Translational Medicine (Partners Board Member), Wistar Institute (Board Member), and the National Foundation of Emergency Medicine (President and Board Member). Dr. Kraft reports grants or contracts from the National Institutes of Health, American Lung Association, Areteia, AstraZeneca, and Sanofi (funds paid to the University of Arizona through June 2022 and now to the Icahn School of Medicine at Mount Sinai); consulting fees from AstraZeneca, Sanofi, Chiesi, GSK, Kinaset, and Genentech for the treatment of asthma and COPD (funds paid personally); honoraria for lectures and presentations from Chiesi, Regeneron, and Genentech (funds paid personally); partial travel support from the European Respiratory Society to attend annual scientific conferences in 2023–2024; one issued and three filed patents related to the development of therapeutics for inflammatory lung disease; leadership or fiduciary roles with the National Heart, Lung, and Blood Advisory Council (completed in 2022) and the Association of Professors of Medicine (ongoing); equity ownership in RaeSedo, Inc. (developing therapeutics for asthma in preclinical stages, no revenue); and serves as a Section Editor for UpToDate for treatment of severe asthma (funds paid personally).

All other authors have reported that they have no relationships relevant to the contents of this paper to disclose.
