# Supplementary material for: Automated diagnosis of chronic obstructive pulmonary disease using deep learning applied to electrocardiograms
Source: eBioMedicine. 2026 Jan 3;123:106066. doi: 10.1016/j.ebiom.2025.106066 (PMC12805298; doi:10.1016/j.ebiom.2025.106066)
Supplement: Supplementary Figures and Tables [file mmc1.docx]

**
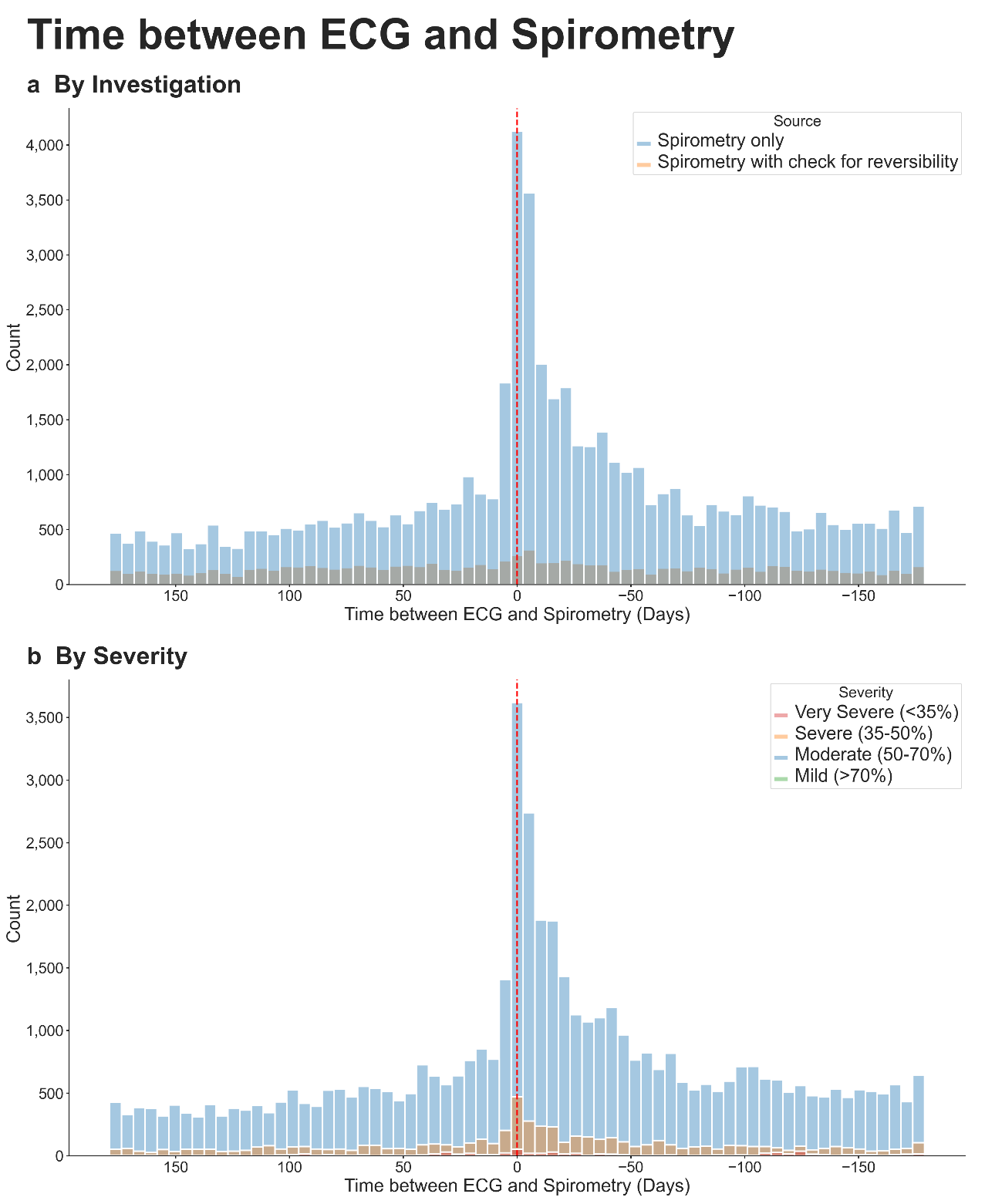
Supplementary Materials**

**Supplementary Figure 1**. **Temporal relationship between date of spirometry and date of ECG collection for pairs of Spirometry and ECG measurements**
Panel a. Check for bronchodilator reversibility was only performed for a subset of patients
Panel b. Gradation of pairs by severity of pulmonary dysfunction as measured by the ratio between Predicted FEV_1_: FEV_1_Predicted FEV_1_ was calculated using the GLI-Global equations.


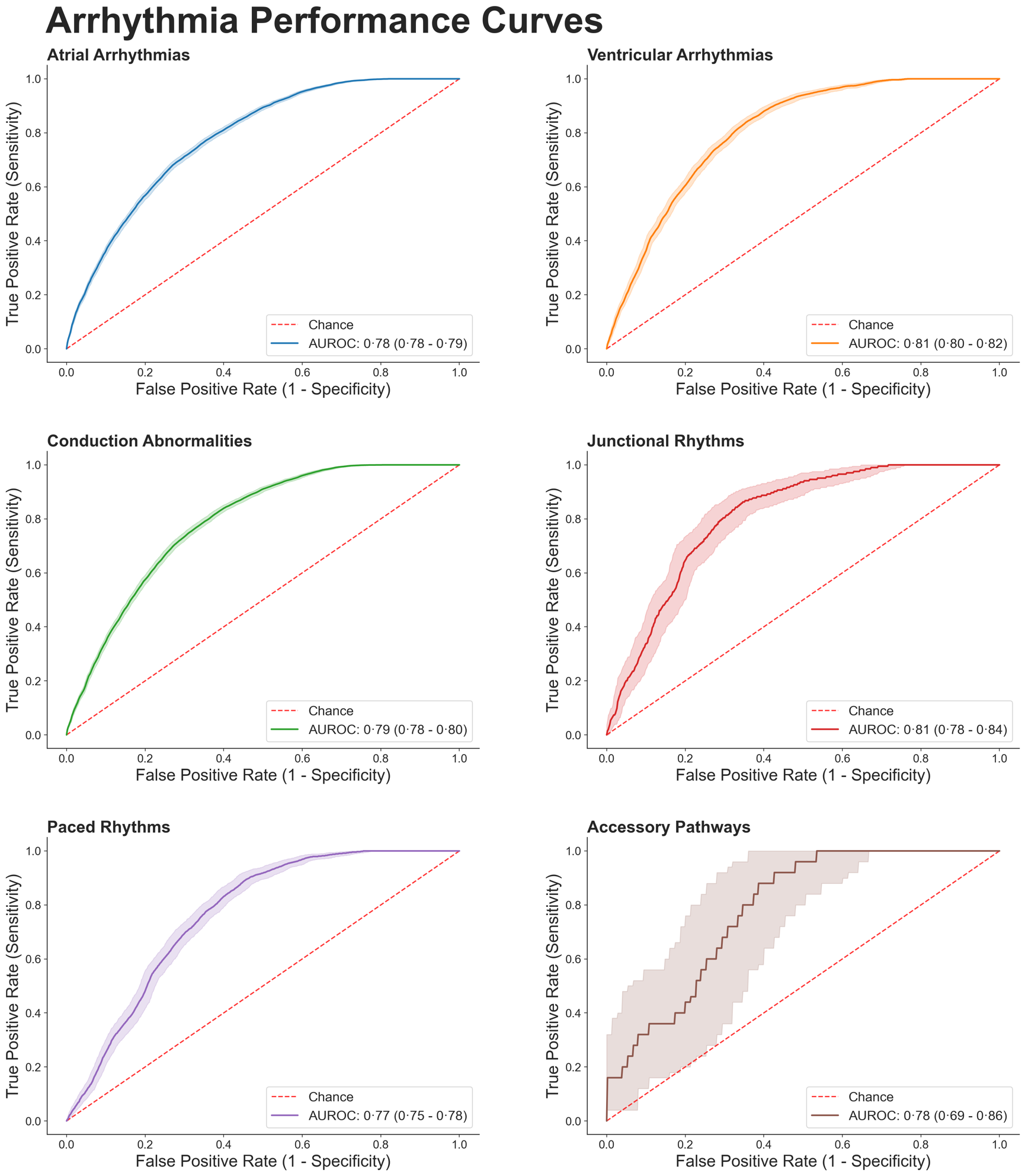


**Supplementary Figure 2**. **Model performance across arrhythmia subtypes**
Receiver Operating Characteristic (ROC) and Precision-Recall (PRC) curves are shown for six clinically relevant arrhythmia groups. Each ROC curve plots the true positive rate (sensitivity) against the false positive rate (1 - specificity). Solid lines represent median performance across 1,000 bootstrap iterations, and shaded bands indicate 95% confidence intervals. Dashed red lines indicate chance-level performance. Discrete steps in certain curves (e.g., Accessory Pathways) reflect limited sample sizes or sparse predicted probability distributions. Area under the ROC (AUROC) values with 95% confidence intervals are reported in the legends.

| **Site** | **Sex** | **AUROC** | **AUPRC** | **Specificity** | **PPV** | **NPV** |
| --- | --- | --- | --- | --- | --- | --- |
| **Internal**  **Testing** | Female | 0⋅80  (0⋅79 - 0⋅81) | 0⋅56  (0⋅55 - 0⋅56) | 0⋅59  (0⋅58 - 0⋅60) | 0⋅44  (0⋅42 - 0⋅43) | 0⋅91  (0⋅91 - 0⋅91) |
|  | Male | 0⋅80  (0⋅80 - 0⋅80) | 0⋅56  (0⋅55 - 0⋅57) | 0⋅58  (0⋅57 - 0⋅59) | 0⋅44  (0⋅44 - 0⋅45) | 0⋅91  (0⋅91 - 0⋅92) |
| **External Validation** | Female | 0⋅82  (0⋅81 - 0⋅82) | 0⋅54  (0⋅54 - 0⋅55) | 0⋅63 (0⋅63 - 0⋅64) | 0⋅41  (0⋅41 - 0⋅42) | 0⋅93  (0⋅93 - 0⋅93) |
|  | Male | 0⋅81  (0⋅81 - 0⋅82) | 0⋅52  (0⋅51 - 0⋅53) | 0⋅62  (0⋅61 - 0⋅63) | 0⋅39  (0⋅37 - 0⋅41) | 0⋅94  (0⋅94 - 0⋅94) |

**Supplementary Table 1. Sex disaggregated metrics of model performance**AUROC and AUPRC are threshold independent metrics. Sensitivity, Specificity, PPV, and NPV are threshold dependent metrics. All threshold dependent metrics assume a sensitivity of 0⋅85.

| Arrhythmia Category | AUROC (95% CI) | Included diagnoses |
| --- | --- | --- |
| Atrial Arrhythmias | 0⋅78 (0⋅78 - 0⋅79) | Atrial Fibrillation, Atrial Flutter, Supraventricular Tachycardia, Supraventricular Tachycardia, Paroxysmal Supraventricular Contraction, Atrial Tachycardia, Atrial Tachycardia, Ectopic Atrial Tachycardia, Ectopic Atrial Rhythm, Ectopic Atrial Rhythm, Retrograde Atrial Activity, Premature Atrial Contraction, Atrial Bigeminy, Atrial Premature Ventricular Contraction, Frequent Premature Atrial Contractions, Wandering Atrial Pacemaker, Sinus Arrest, Sinus Pause, Sinus Bradycardia, Sinus Bradycardia, Sinus Tachycardia, Sinus Arrhythmia |
| Ventricular Arrhythmias | 0⋅81 (0⋅80 - 0⋅82) | Premature Ventricular Contraction, Frequent PVCs, Ventricular Tachycardia, Nonsustained Ventricular Tachycardia, Ventricular Fibrillation, Idioventricular Rhythm, Ventricular Escape Rhythm, Wide Complex Tachycardia, Junctional Ectopic Tachycardia, Fusion Beats |
| Conduction Abnormalities | 0⋅79 (0⋅78 - 0⋅80) | Right Bundle Branch Block, Left Bundle Branch Block, Bifascicular Block, Trifascicular Block, Intraventricular Conduction Delay, First-Degree AV Block, Second-Degree AV Block, Mobitz I Second-Degree AV Block, Mobitz II Second-Degree AV Block, Third-Degree AV Block, AV Block (unspecified) |
| Junctional Rhythms | 0⋅81 (0⋅78 - 0⋅84) | Junctional Rhythm, Junctional Rhythm, Junctional Bradycardia, Junctional Tachycardia, Premature Junctional Contraction |
| Paced Rhythms | 0⋅77 (0⋅75 - 0⋅78) | Paced Rhythm, Ventricular Paced Rhythm, Biventricular Paced Rhythm, Atrial Paced Rhythm, Ventricular Pacemaker, Atrial Pacemaker, Pacemaker Rhythm, Atrioventricular Sequential Pacing, Left Ventricular Paced Rhythm, Biventricular Pacing Check, Atrial and Ventricular Pacing |
| Accessory Pathways | 0⋅78 (0⋅73 - 0⋅83) | Wolff-Parkinson-White Syndrome, Aberrant Conduction, Retrograde P Waves, Retrograde Atrial Activity |

**Supplementary Table 2. Model performance across arrhythmia subtypes**Each row summarizes the area under the receiver operating characteristic curve (AUROC) with 95% confidence intervals for a predictive model evaluated on a group of related arrhythmias. The rightmost column lists the specific rhythm diagnoses included in each category, with abbreviations expanded to full clinical terminology for clarity.
